# A systematic review and meta-analysis reveals enrichment of pro-inflammatory and bacterial immunity-modulating taxa in oral squamous cell carcinoma

**DOI:** 10.3389/fimmu.2026.1913744

**Published:** 2026-09-15

**Authors:** Akanksha Vishwakarma, Ayurshi Patil, Diksha Saini, Akshay Shankar, Satyendra Kumar Shukla, Keerti Kushwaha, Manupal Choudhary, Manu Bhardwaj, Vandna Gupta, Chander Prakash Yadav, Pramod Kumar

**Affiliations:** 1 Division of Molecular Biology, Indian Council of Medical Research (ICMR)-National Institute of Cancer Prevention and Research, Noida, India; 2 Academy of Scientific and Innovative Research (AcSIR), AcSIR Headquarters CSIR-HRDC Campus, Ghaziabad, Uttar Pradesh, India; 3 Department of Microbiology, Chaudhary Charan Singh University, Meerut, India; 4 Indian Council of Medical Research (ICMR)-National Institute of Cancer Prevention and Research (NICPR), IT-Cell, Noida, India; 5 Division of Epidemiology and Biostatistics, Indian Council of Medical Research (ICMR)-National Institute of Cancer Prevention and Research (NICPR), Noida, India

**Keywords:** conserved taxa, geography, NGS, oral microbiome, OSCC

## Abstract

**Introduction:**

Oral squamous cell carcinoma (OSCC) is a major concern due to high recurrence, therapeutic resistance, and mortality. Increasing evidence indicates that oral microbiome dysbiosis contributes to OSCC development, yet comprehensive multi-cohort evaluations are limited. A systematic review and meta-analysis of the oral microbiome was conducted to identify conserved taxa in patients with OSCC.

**Methods:**

A systematic review and meta-analysis was conducted according to PRISMA guidelines, using data from PubMed, Embase, Web of Science, and Google Scholar. Raw 16S rDNA sequences were processed in QIIME 2 (v2024.10) for quality control, taxonomic classification, and diversity analysis. Linear Discriminant Analysis Effect Size (LEfSe) analyses and machine learning were used for the identification of conserved taxa.

**Results:**

Significant microbial shifts were observed between OSCC and healthy groups. OSCC samples showed enrichment of taxa, which further identified as conserved taxa such as *Porphyromonas*, *Fusobacterium*, *Campylobacter*, *Catonella*, *Prevotella*, *Selenomonas*, and *Veillonella*, with species including *Campylobacter showae*, *Capnocytophaga granulosa*, *Capnocytophaga leadbetteri*, *Prevotella loescheii*, *Streptococcus anginosus*, *Streptococcus pneumoniae*, and *Treponema medium*. Healthy individuals exhibited higher levels of *Actinobacillus*, *Corynebacterium*, *Escherichia-Shigella*, and *Haemophilus*, particularly *H. parainfluenzae*, *Rothia aeria*, *R. dentocariosa*, and *Selenomonas* spp. Bray–Curtis beta-diversity analysis confirmed significant compositional differences between groups. Sample type and geography-based comparisons revealed additional variation: Geographically, *Lautropia* and *Haemophilus* were common across regions, whereas *Cutibacterium*, *Escherichia-Shigella*, *Gracilibacteria*, and several species appeared specific to India. Functional predictions revealed enrichment of pathways related to environmental and genetic information processing, as well as cellular processes in OSCC. Notably, significant enrichment of genes involved in inflammation, ABC transporters, and bacterial immunity was observed in OSCC groups.

**Conclusion:**

Overall, this study demonstrates the enrichment of microbial taxa associated with chronic inflammation and carcinogenesis, along with a depletion of protective commensal microbes in patients with OSCC. These findings highlight distinct taxonomic and functional microbiome signatures associated with OSCC and emphasize the influence of sample type and geography on oral microbial composition. The enrichment of predicted inflammatory genes, transport-associated genes, and bacterial immune-related genes suggests the potential contribution of microbial communities to chronic inflammation and tumor progression.

## Introduction

According to the GLOBOCAN report by IARC in 2022, lip and oral cavity cancers rank 16th in incidence and 15th in mortality (1). These cancers accounted for 389,485 cases and 188,230 deaths worldwide. Approximately 90% of oral cavity cancers are classified as oral squamous cell carcinoma (OSCC) (2). Various risk factors associated with OSCC include smoking, alcohol consumption, periodontal disease, genetic predisposition, and the microbiome (3, 4).

The oral cavity is considered one of the largest reservoirs of various microorganisms, including various species of bacteria, fungi, and viruses (5). The microbiome’s associative roles have been established in clinical outcomes as improved antitumor immune response and anti-proliferative effect in oral cancer (OC) (6). By producing carcinogenic compounds (such as cytolethal distending toxin, typhoid toxin, nitrosamine, and colibactin) that target DNA and cause mutations, pro-inflammatory reactions, and direct proliferative effects on cells in the oral epithelium, the oral microbiome may be responsible for OC. Reactive oxygen species (ROS) produced by bacteria interacting with fibroblasts and immune cells can also cause DNA damage in epithelial cells (7).

Despite the associative roles of microbiota, a direct validation in improving oral hygiene for anti-proliferative efficacy needs to be established. It has been observed that pathobionts found in the oral cavity are associated with cancer progression, including OSCC (8). Several metagenomic studies of the microbiome have identified changes in microbial patterns in OC, which differ further based on the stage of OC, malignant lesions, and oral cavity disorders (7). In addition to dysbiosis in OC, investigations also suggest bacterial alterations due to tobacco chewing, which increases a person’s susceptibility to bacterial infections by causing bacterial virulence, disrupting host immune responses, and altering the structure and physiology of the human oral cavity (9).

Diversity of the oral bacteriome translates to its bacterial genus diversity, which is seen conserved as well as varying to a great degree in an individual with respect to niches, individuals, demography, age, lifestyle choices, diet, etc. in both healthy and diseased states (10). The various modifiable and unmodifiable risk factors for OC (alcohol, host genetics, microbial infection, nutritional pattern, and tobacco) also shape the oral microbiota, its diversity/richness, and dysbiosis. Despite HPV, periodontal pathobionts such as *Fusobacterium nucleatum* (Gram-negative) and *Porphyromonas gingivalis* are important risk factors for OSCC, and their associative role in OSCC progression has been established (11). Their presence in the tumor microenvironment has also been documented (8, 12). Recent *in vitro* studies prove a direct role in metastasis, as both *F. nucleatum* and *P. gingivalis* are independently capable of converting epithelial to p-EMT phenotype in OSCC cells (13). Some oral microbes provide protective efficacy against OSCC by inhibiting the colonization of pro-cancer pathobionts in the oral cavity (14).

A study from Bhopal, central India, suggested the enrichment of *Capnocytophaga*, *Fusobacterium*, and *Prevotella* in the oral microbiome as potential microbiome marker genera for OC (15), contrary to an earlier report (16). A study from Chennai reported an enrichment of *Alloscardovia*, *Capnocytophaga*, *Fusobacterium*, *Morganella*, *Propionibacterium*, and *Pseudomonas* in patients with OC, whereas *Corynebacterium*, *Prevotella*, and *Streptococcus* were identified as microbial markers associated with healthy controls (17). In contrast, *Corynebacterium* and *Prevotella* were found to be enriched in patients with OC in Kolkata and Bhopal (15, 18). Overall, there is a lack of comprehensive, consistent, and standardized evidence defining a distinct microbial signature in the oral microbiome of patients with OSCC (19). The observed heterogeneity across studies may be attributed to differences in sample collection, sequencing and data-generation methods, microbial taxonomic assignment, and analytical approaches. Therefore, we conducted a systematic review and meta-analysis to synthesize the available evidence using a standardized approach to microbial taxonomic assignment, thereby minimizing methodological heterogeneity and enabling a more robust characterization of microbial signatures associated with OSCC.

## Methods

This systematic review and meta-analysis, conducted and reported in accordance with the PRISMA guidelines (20), aimed to systematically synthesize the available evidence on differences in the composition and diversity of the oral microbiota between patients with OSCC and healthy controls, and to identify microbial taxa potentially associated with OSCC.

### Literature search and search term

A comprehensive search strategy was developed based on relevant literature and applied across four electronic databases: PubMed, Embase, Web of Science, and Google Scholar. The search was restricted to articles published in the English language and included all eligible studies published from database inception through 1 August 2024.

### Eligibility criteria

#### Inclusion criteria

Studies were eligible for inclusion if they investigate the oral bacterial microbiome in patients with OSCC and/or healthy controls using human-derived oral samples, including saliva, oral tissue, swabs, dental plaque, or oral rinse samples. Studies were required to employ 16S rRNA gene-based next-generation sequencing (NGS) and have publicly available raw sequencing data (SRA files) in the NCBI Sequence Read Archive, with an associated BioProject ID. To ensure data quality and comparability for downstream analysis, only datasets comprising paired-end sequencing reads and meeting the predefined quality-control threshold (QC >30) were included.

#### Exclusion criteria

Review articles, systematic reviews, meta-analyses, and other non-original research articles were excluded. Studies were also excluded if they were outside the scope of the review; lacked a healthy control group and instead used adjacent non-tumor tissue cells (ANTCs) as a comparator; and focused on oral potentially malignant disorders (OPMDs), oropharyngeal cancer, salivary adenoid cystic carcinoma, mRNA-based metatranscriptomics, or the mycobiome. Additionally, studies for which raw sequencing data were not publicly available in the specified repository were excluded.

#### Selection process

Based on predefined inclusion and exclusion criteria, all the articles retrieved from the above given four databases were initially screened by title and abstract, followed by full-text screening. The screening process was independently conducted by two reviewers (AP and DS). Any disagreements regarding study eligibility were resolved through discussion and, when necessary, consultation with a third reviewer (PK). Detailed search strategy specific to search.

#### Data extraction

Data extraction was performed using a structure sheet in MS Excel by the two reviewers (AP and DS) independently and cross-checked by a third reviewer (PK).

#### Taxonomic meta-analysis

To examine the microbial diversity and taxonomic composition at the phylum, genus, and species levels, raw sequence data corresponding to the BioProject IDs of included studies were retrieved and downloaded from the European Nucleotide Archive (ENA) (https://www.ebi.ac.uk/ena/browser/home). Raw sequence reads were processed using QIIME 2 (Quantitative Insights into Microbial Ecology) pipeline (ver. 2024.10). Sequence quality filtering, denoising, and chimera removal were performed using the q2-dada2 plugin. Taxonomy assignment of high-quality sequence reads was performed using the QIIME 2 feature classifier plugin against the SILVA 16S database (21). Following bioinformatic processing, unclassified taxa and taxa with zero counts were excluded at the phylum, genus, and species levels. The resulting taxonomic profiles were further filtered to retain taxa with a relative abundance greater than 0.05% for downstream analyses.

### Diversity analysis and functional prediction

Microbial diversity was assessed using multiple alpha- and beta-diversity metrics implemented in the QIIME 2 pipeline. Alpha-diversity was evaluated using the Chao1, Shannon, Simpson, PD whole tree, and Good’s coverage estimator. Beta-diversity was assessed using Bray–Curtis dissimilarity and weighted UniFrac distance. The putative functional prediction of bacterial diversity of these studies was analyzed using the PICRUSt2 (v 2.4.1) tool (22). Representative sequences of amplicon sequence variants (ASVs) and the corresponding biome table of ASVs across samples were used as input into the PICRUSt2 tool.

### Statistical analysis

Relative abundance data at three taxonomic levels (phylum, genus, and species) were extracted from the included studies using the bioinformatics pipelines described above and analyzed separately at each level. To standardize comparisons across studies, relative abundance at each taxonomic level was expressed as a percentage of total reads within an individual. Taxa (phyla, genera, or species) present at a mean relative abundance of <1%, as well as those with inconsistent or irregular reporting across studies, were excluded from further analysis to minimize noise from low-prevalence or unreliably quantified taxa. Percentage relative abundance was compared between the OSCC and healthy control groups using the Mann–Whitney *U* test, given the non-normal distribution of relative abundance data typical of microbiome datasets, and results were expressed as median (interquartile range, P25–P75). All statistical analysis was performed in Statistical software R 4.6.1. The differentially expressed taxa with statistical significance were further subjected to calculate the effect size using Linear Discriminant Analysis Effect Size (LEfSe) analysis.

Machine learning analyses were conducted in Python 3.13.9 using the H2O module (v3.38.0.2). The Gradient One-Side Sampling (GOSS) algorithm within the LightGBM framework was applied because of its efficiency with high-dimensional and imbalanced datasets and its built-in feature importance capability. Model performance was evaluated using fivefold cross-validation and external validation with accuracy, AUC, F1-score, precision, and recall. Hyperparameter tuning was performed, and the top 20 features were identified as potential diagnostic biomarkers. Furthermore, R (version 3.6.0) was used for generating box plots, forest plots, functional prediction analyses, and diversity indices. Heatmaps were prepared using Morpheus Broad Institute software (https://software.broadinstitute.org/morpheus/). Python coding language was used for data interpretation of PICRUSt-based functional gene analysis.

## Results

The initial searches yielded studies. Only 11 studies were found eligible based on the inclusion criteria after final qualitative synthesis and data extraction (metadata and NGS data). A summary of the search and selection process being used in the study is outlined in **Figure 1**.

**Figure 1:**
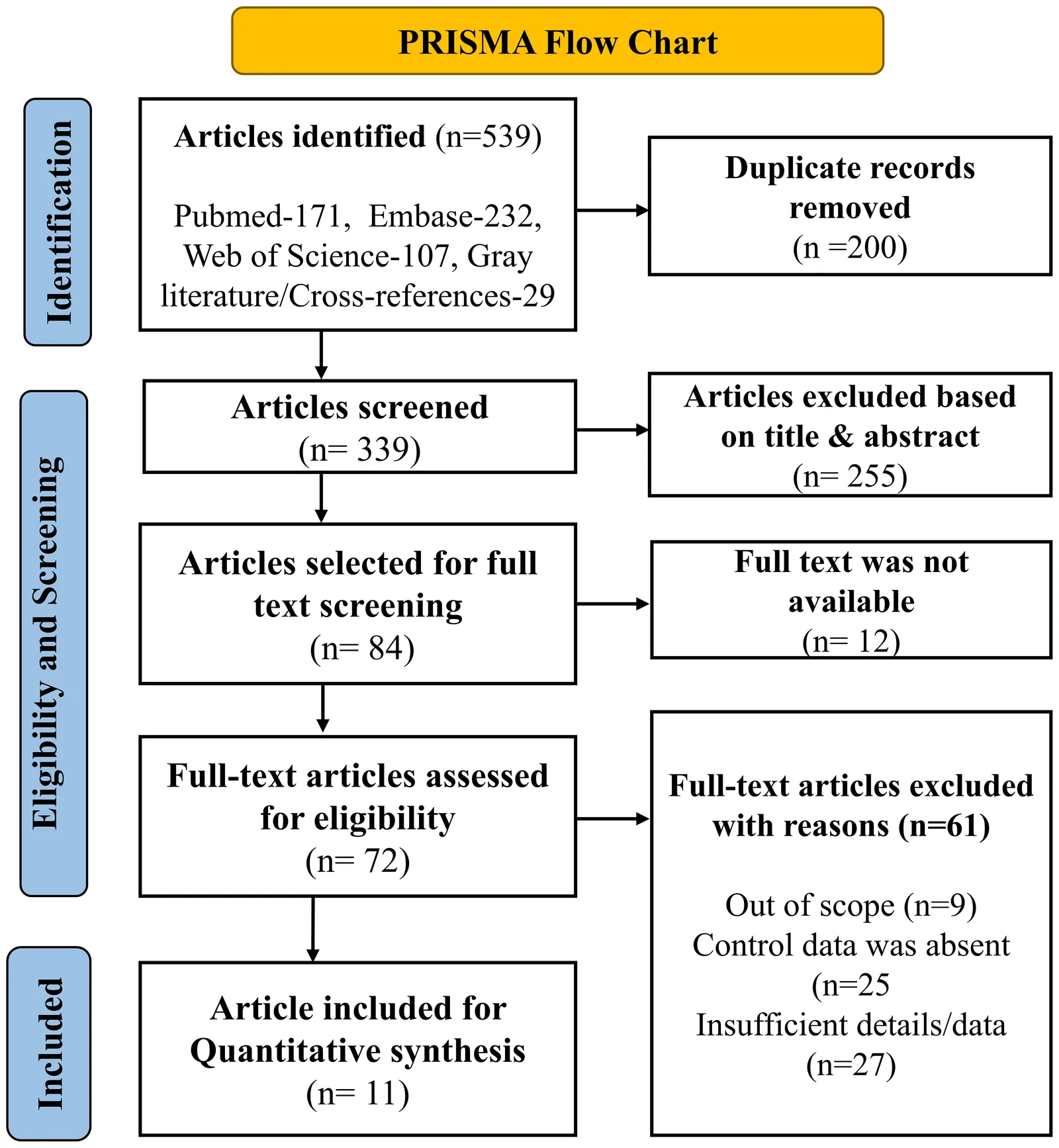
The PRISMA flow diagram summarizes the systematic literature search and study selection process. Of the identified studies, only 11 studies met the predefined inclusion criteria for meta-analysis and provided publicly accessible datasets.

### Diversity indices

Meta-analysis of alpha-diversity, which reflects the within-sample richness and diversity of the oral microbiome, showed no significant differences between the OSCC and healthy control groups across all evaluated indices. Shannon diversity was comparable between the two groups (Heterogeneity: *I*² = 83.6%; SMD = −0.10, 95% CI: −0.45 to 0.26) (**Figure 2A**), indicating no significant difference in microbial richness and evenness. Similarly, observed features (*I*² = 98.1%; SMD = −37.18, 95% CI: −78.96 to 4.61), Faith’s phylogenetic diversity (*I*² = 91.9%; SMD = −5.51, 95% CI: −22.64 to 11.63), evenness (*I*² = 75.9%; SMD = −0.01, 95% CI: −0.02 to 0.03), and Chao1 richness (*I*² = 91.3%; SMD = −50.39, 95% CI: −101.73 to 0.96) also did not differ significantly between the two groups, suggesting that the overall richness, phylogenetic diversity, and evenness of the oral microbial communities were largely preserved in OSCC, while meta-analysis of beta-diversity, which assesses differences in microbial community composition between samples, demonstrated a significant difference between the OSCC and healthy control groups based on the Bray–Curtis distance matrix (Heterogeneity: *I*² = 81.8%; SMD = 1.29, 95% CI: 0.68 to 1.90) (**Figure 2B**). In contrast, the Weighted UniFrac distance matrix did not demonstrate a statistically significant difference (Heterogeneity: *I*² = 75.2%; SMD = 1.26, 95%; CI: −0.47 to 2.98). Stratification by sample type and geographic region did not reveal significant differences in alpha-diversity between healthy controls and patients with OSCC. Conversely, Bray–Curtis beta-diversity showed significant differences across both subgroup analyses, suggesting that microbial community composition, rather than within-sample diversity, differs between OSCC and healthy individuals (**Supplementary Figures 1–19**).

**Figure 2:**
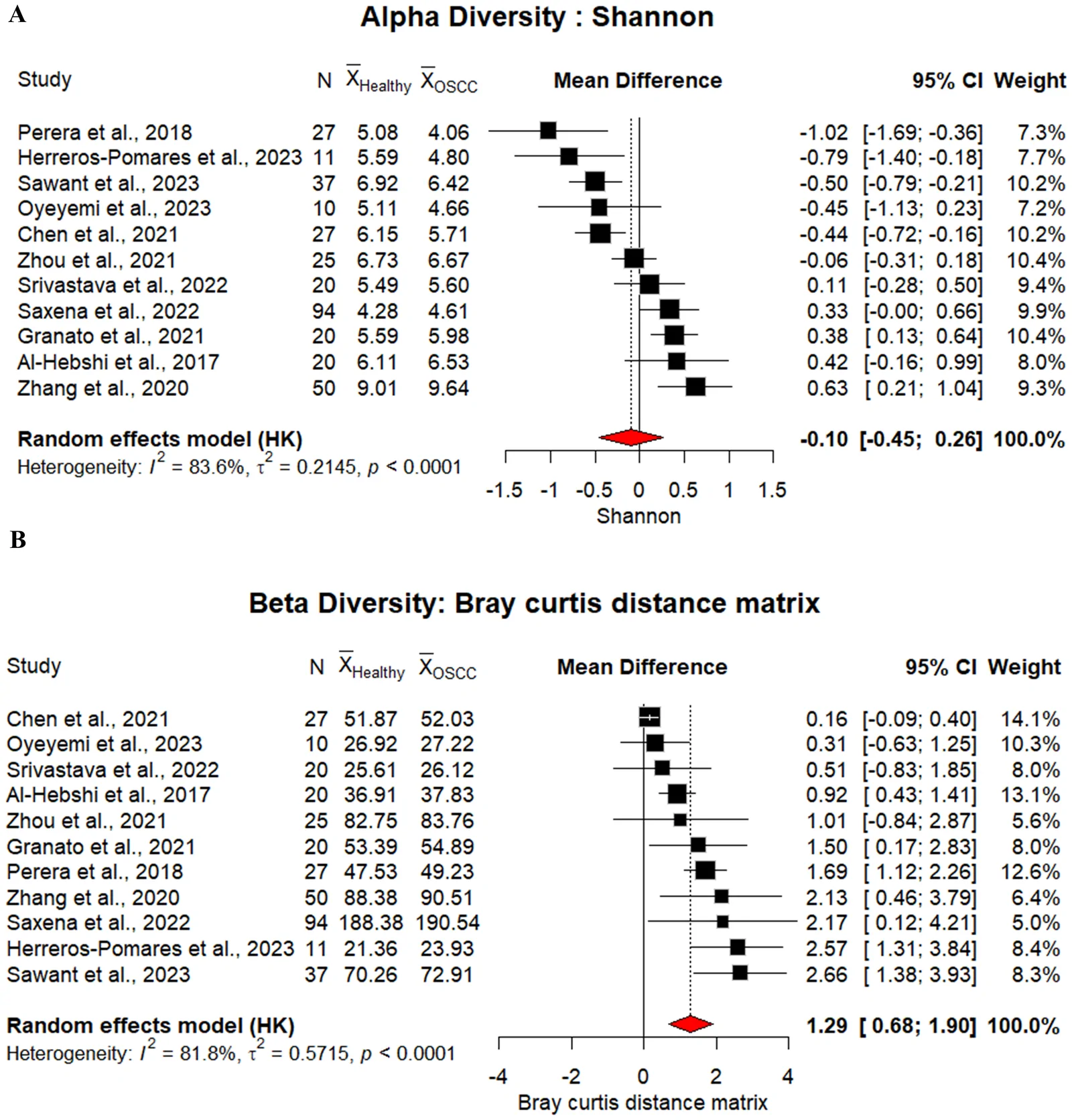
Forest plots represent the pooled effect size of alpha (Shannon) **(A)** and beta (Bray–Curtis) diversity **(B)** of multiple studies for meta-analysis using the random effects model (HK) with 95% CI.

### Microbiome taxonomy at different levels of taxa (phylum, genus, and species)

The phylum-level taxonomy analysis identified 11 different phyla with relative abundance ≥0.05% after excluding unclassified and zero-count phyla. Among these, eight phyla (Actinobacteriota, Bacteroidota, Campilobacterota, Cyanobacteria, Firmicutes, Fusobacteriota, Proteobacteria, and Spirochaetota) showed a statistically significant difference between the healthy and OSCC groups among the included studies. Of these phyla, six different phyla (Bacteroidota, Campilobacterota, Firmicutes, Fusobacteriota, Proteobacteria, and Spirochaetota) were significantly high in OSCC groups, while two different phyla, Actinobacteriota and Cyanobacteria, were significantly high in healthy groups. At the genus level, a total of 162 genera with a relative abundance of ≥0.05% were identified among both groups. However, 79 genera were selected for the analysis of differential abundance based on their presence in more than 50 individuals from both the healthy and OSCC groups. The statistically significant difference in the abundance was observed in 29 genera between the healthy and OSCC groups. Twenty genera, including *Absconditabacteriales* (SR1), *Aggregatibacter*, *Campylobacter*, *Catonella*, *Filifactor*, *Peptoaerobacter*, *Prevotella*, *Pseudopropionibacterium*, and *Treponema*, were significantly enriched in patients with OSCC, while nine genera including *Actinobacillus*, *Corynebacterium*, *Escherichia-Shigella*, *Gracilibacteria*, *Haemophilus*, and *Lautropia* were significantly abundant in healthy groups.

A total of 300 species with a relative abundance of ≥0.05% were identified, and 108 species were selected for further analysis to calculate differential abundance as previously described. Among these, 33 species showed a significant difference in abundance between the healthy and OSCC groups. Of the 28 species identified, the following exhibited the highest significance: *Capnocytophaga sputigena*, *Lachnospiraceae bacterium*, *Haemophilus* sp., *Prevotella loescheii*, *S. anginosus*, *S. pneumoniae*, *Treponema denticola*, *Treponema lecithinolyticum*, *Capnocytophaga leadbetteri*, *Dialister pneumosintes*, and *Peptostreptococcus stomatis*. Additionally, some rarer species such as *Capnocytophaga granulosa*, *Catonella* genomosp., *Phocaeicola abscessus*, *Prevotella baroniae*, *Solobacterium moorei*, *Treponema lecithinolyticum*, *Treponema medium*, *Peptostreptococcus stomatis*, and *Dialister pneumosintes* also showed significant abundance in the OSCC group. However, *Haemophilus parainfluenzae*, *Rothia aeria*, *Rothia dentocariosa*, *Rothia mucilaginosa*, and *Selenomonas* species were found to be inhibited in the OSCC group.

### Impact of various non-modifiable risk factors (sample type and geography) on microbiome abundance

Sample type analysis showed the highest number of genera and species observed in tissue samples (*n* = 10 and *n* = 15). The impact of various geographical differences was also observed in terms of the taxa abundances. China had the highest number of significantly different species (*n* = 29), while India had the highest number of significantly different genera (*n* = 12). Sri Lanka had the fewest significant differences at both levels (one genus and two species).

At the genus level, *Actinobacillus* and *Lautropia* were enriched in dental plaque and tissue samples from healthy individuals. In contrast, *Pseudopropionibacterium*, *Tannerella*, and *Treponema* were predominantly enriched in dental plaque samples of patients with OSCC, whereas *Aggregatibacter*, *Bergeyella*, and *Catonella* showed higher abundance in OSCC tissue samples. Furthermore, *Butyrivibrio*, *Lautropia*, and *Mogibacterium* were significantly dominant in saliva samples from patients with OSCC. Genera such as *Bacteroides* and *Phocaeicola* were enriched in OSCC groups, whereas *Corynebacterium*, *Haemophilus*, and *Tannerella* were more abundant in healthy controls of oral rinse samples (**Table 1**; **Figure 3A**). Species-level analysis revealed that *C. showae*, *C. granulosa*, *C. leadbetteri*, *C. sputigena*, *C.* genomosp., *L. bacterium*, *N. perflava*, and *T. medium* were significantly enriched in the OSCC group, while *H. parainfluenzae*, *R. mucilaginosa*, *S. pneumoniae*, *T. forsythia*, *Tannerella* sp., *T. maltophilum*, and *T. socranskii* were enriched in the healthy group of tissue samples. *C. bacterium*, *D. pneumosintes*, *Haemophilus* sp., *M. pumilum*, *P. oris*, *P. veroralis*, *S. odontolytica*, *S. sputigena*, *S. anginosus*, *S. infantis*, *T. forsythia*, and *T. lecithinolyticum* showed dominancy in the OSCC group of saliva samples. *C. concisus*, *C. leadbetteri*, *C. sputigena*, *D. pneumosintes*, *Haemophilus* sp., *Metamycoplasma salivarium*, *Parvimos micra*, *P. stomatis*, and *S. moorei* enriched oral rinse in the OSCC group (**Table 1**; **Figure 3B**).

**Table 1:** Significant Abundance of taxa at the genus and species level: sample type and country-specific taxa.

Factor	Group	Genus	Species
Sample type	Tissue	Healthy- *Actinobacillus, Corynebacterium* OSCC- *Aggregatibacter, Bergeyella*, *Catonella, Lachnoaerobaculum, Lautropia, Lentimicrobium, Peptoaerobacter*	Healthy- *H. parainfluenzae, R. mucilaginosa, S. pneumoniae, T. forsythia, Tannerella sp, T. maltophilum*, and *T. socranskii* OSCC- *C. showae, C. granulosa, C. leadbetteri, C. sputigena, C. genomosp.*, *L. bacterium, N. perflava, T. medium*
	Saliva	OSCC- *Butyrivibrio, Lautropia*, and *Mogibacterium*	Healthy- *C. concisus, Leptotrichia sp.* OSCC- *C. bacterium, D. pneumosintes, Haemophilus_sp*, *M. pumilum, P. oris, P. veroralis, S. odontolytica, S. sputigena, S. anginosus, S. infantis, T. forsythia*, and *T. lecithinolyticum*
	Oral rinse	Healthy- *Corynebacterium, Haemophilus, Tannerella* OSCC- *Bacteroides, Phocaeicola*	Healthy- *C. durum, D. invisus, H. parainfluenzae, R. dentocariosa, S. parasanguinis* OSCC- *C. concisus, C. leadbetteri, C. sputigena, D. pneumosintes, Haemophilus sp., Metamycoplasma salivarium, Parvimos micra, P. stomatis, S. moorei*
	Dental plaque	Healthy- *Actinobacillus, Lautropia, Escherichia-Shigella, Haemophilus* OSCC- *Pseudopropionibacterium, Tannerella*, and *Treponema*	--
Geography	India	Healthy- *Absconditabacteriales (SR1), Actinobacillus, Corynebacterium, Cutibacterium, Escherichia-Shigella, Gracilibacteria, Haemophilus, Kingella, Lautropia, Megasphaera, Prevotella*, and *Selenomonas* OSCC- 0	Healthy- *P. melaninogenica, S. parasanguinis, T. denticola, H. parainfluenzae, C. durum, H. sputorum*, and *Porphyromonas-like sp*. OSCC- *C. sputigena, Haemophilus sp., C. leadbetteri, P. stomatis, D. pneumosintes, C. concisus, M. salivarium.*
	China	Healthy- 0 OSCC- *Catonella, Filifactor, Haemophilus, Lachnoaerobaculum, Lautropia, Lentimicrobium, Peptoaerobacter, Phocaeicola, Pseudopropionibacterium, Selenomonas*, and *Treponema*	Healthy- *R. aeria, R. dentocariosa, S. pneumoniae, T. forsythia*, OSCC- *C. showae, C. sputigena, C. genomosp., L. bacterium, P. stomatis, P. abscessus, P. baroniae, P. intermedia, P. loescheii, P. melaninogenica, S. moorei, S. anginosus, T. denticola, T. lecithinolyticum, T. socranskii, C. leadbetteri, H. parainfluenzae, P. stomatis, D. pneumosintes, T. maltophilum, L. shahii, N. elongata, P. nceiensis, S. infantis*, and *Tannerella sp*
	Yemen	Healthy- *Haemophilus, Lautropia* OSCC- *Campylobacter, Catonella, Mogibacterium, Prevotella*, and *Treponema*	Healthy- *R. dentocariosa* *C. granulosa, S. sputigena, S. parasanguinis, H. parainfluenzae, S. infantis*, and *V. dispar*
	Taiwan	Healthy- *Corynebacterium, Kingella, Lachnoaerobaculum* OSCC- *Lautropia*, *Mogibacterium*	Healthy- *M. pumilum, Selenomonas sp., C. matruchotii, C. concisus, P. jejuni, P. oulorum* OSCC- *C. sputigena, T. lecithinolyticum, P. stomatis, T. forsythia*
	Spain	Healthy- *Tannerella* OSCC- 0	Healthy- *D. invisus, T. forsythia*, and *T. maltophilum* OSCC- *P. melaninogenica, P. pasteri*
	Sri Lanka	Healthy- *Actinobacillus*	Healthy- *R. mucilaginosa* and *L. wadei*

**Figure 3:**
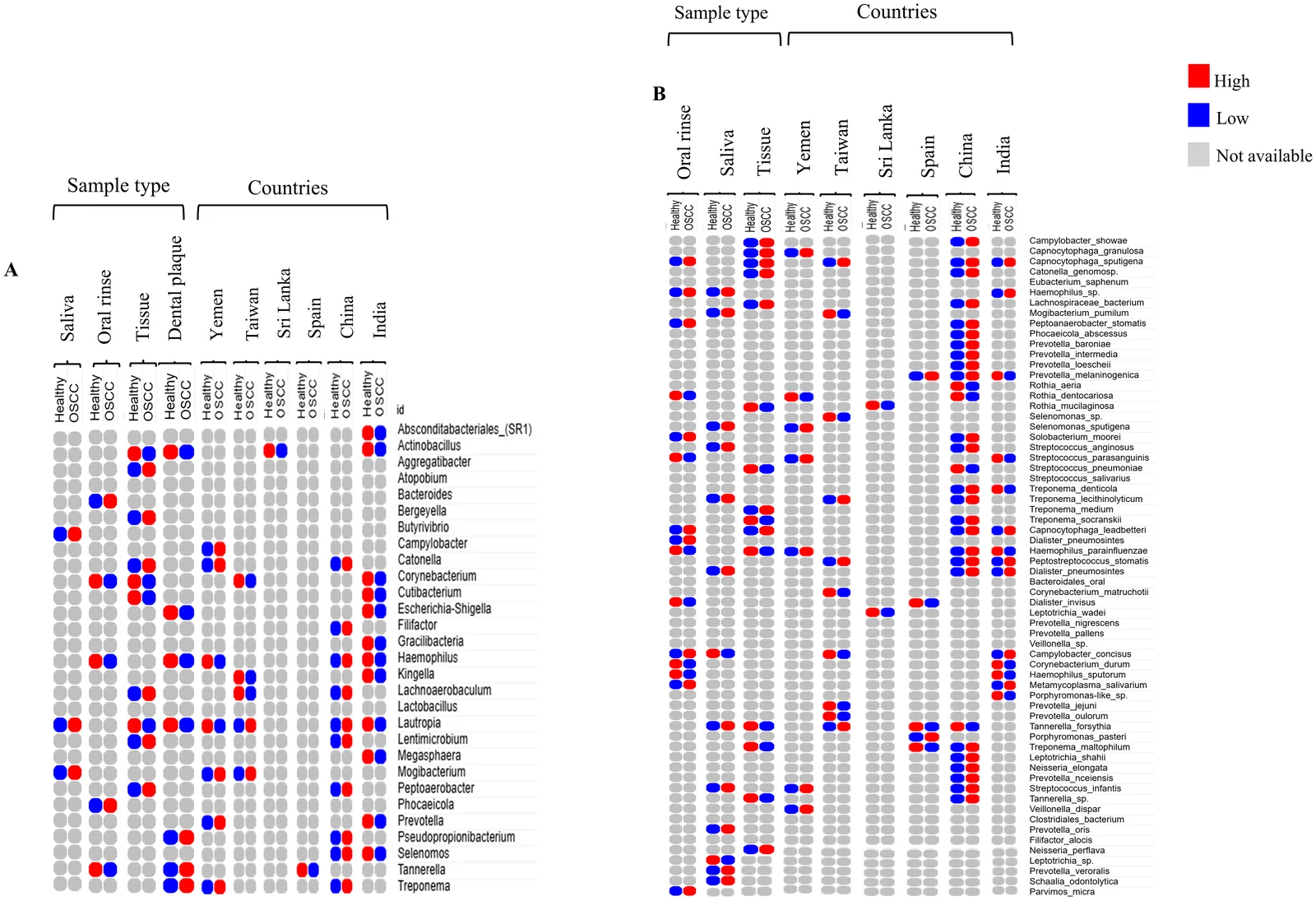
**(A)** Heatmap represents the highest significant difference at the genus level with age, sample type, and country. **(B)** The highest significant difference at the species level with age, sample type, and country.

The genus *Lautropia* was found in four countries; in India and Yemen, they are found in healthy controls, and in China and Taiwan, they are found in OSCC groups. *Haemophilus* was highly abundant in healthy individuals in India and Yemen and patients with OSCC in China. Additionally, the genera *Corynebacterium* and *Kingella* were present in healthy individuals in both India and Taiwan, while *Catonella* and *Treponema* were found in patients with OSCC in Yemen and China. The genus *Actinobacillus* is commonly abundant in healthy groups in India and Sri Lanka. *Lachnoaerobaculum* showed dominance in the OSCC group in China and healthy individuals in Taiwan. *Mogibacterium* was common in patients with OSCC in Yemen and Taiwan. *Prevotella* and *Selenomonas* were significantly high in India’s healthy individuals, and *Prevotella* is highly abundant in patients with OSCC in Yemen while *Selenomonas* was high in patients with OSCC in China. Some genera were specifically found only in one country, such as *Absconditabacteriales* (SR1), *Cutibacterium*, *Escherichia-Shigella*, *Gracilibacteria*, and *Megasphaera*, which were unique to healthy individuals in India, while *Filifactor*, *Lentimicrobium*, *Peptoaerobacter*, *Phocaeicola*, and *Pseudopropionibacterium* were specifically present in patients with OSCC in China. Finally, the genera *Campylobacter* and *Tannerella* were present in patients with OSCC in Yemen and healthy individuals in Spain, respectively (**Table 1**; **Figure 3A**).

Furthermore, when examining differences by country, *P. melaninogenica* were significantly present in three different countries: in Spain and China (patients with OSCC) and in India (healthy individuals). *T. forsythia* was significantly common in healthy individuals in Spain and China, and in Taiwan, it is present in patients with OSCC. *P. stomatis was* significantly found in patients with OSCC in India, Taiwan, and China. *C. leadbetteri* and *D. pneumosintes* were commonly significant in patients with OSCC in India and China. Both *T. denticola* and *H. parainfluenzae* species were abundant in healthy individuals in India but enriched in patients with OSCC in China. *C. sputigena* and *T. lecithinolyticum* were significantly enriched in patients with OSCC in Taiwan and China. *S. infantis* was prevalent in patients with OSCC in China and Yemen, and *R. dentocariosa* was prevalent in healthy individuals in China and Yemen, while *C. concisus* was commonly found in healthy groups in Taiwan and India. A large group of species, including *C. showae*, *C.* genomosp., *L. bacterium*, *P. stomatis*, *P. abscessus*, *P. baroniae*, *P. intermedia*, *P. loescheii*, *S. moorei*, *S. anginosus*, *T. socranskii*, *T. maltophilum*, *L. shahii*, *N. elongata*, *P. nanceiensis*, and *Tannerella* sp., were uniquely present in patients with OSCC, and *R. aeria* and *S. pneumoniae* were present in healthy individuals in China. Conversely, *S. parasanguinis*, *C. durum*, *H. sputorum*, and a *Porphyromonas*-like species were significantly enriched in healthy individuals, while *C. sputigena*, *Haemophilus* sp., and *M. salivarium* were enriched in the OSCC group in India. *M. pumilum*, *Selenomonas* sp., *C. matruchotii*, *P. jejuni*, and *P. oulorum* were abundant in healthy individuals in Taiwan. In Yemen, *C. granulosa*, *S. sputigena*, *S. parasanguinis*, and *V. dispar* were present in the OSCC group. *D. invisus* and *T. maltophilum* were significantly abundant in healthy individuals, *P. pasteri* was abundant in healthy individuals in Spain, while *R. mucilaginosa* and *L. wadei* were observed in the healthy group in Sri Lanka (**Table 1**; **Figure 3B**).

### Composition of core microbiome among the studies

Seven phyla (Firmicutes, Bacteroidota, Proteobacteria, Fusobacteria, Actinobacteriota, Campilobacterota, and Spirochaetota) were present among all the eligible studies. Firmicutes was observed as the most dominant phylum in seven healthy groups (studies 1, 2, 3, 8, 9, 10, and 11) and seven OSCC groups (studies 2, 3, 6, 8, 3, 10, and 11). Proteobacteria was found to be abundant in the four healthy groups (studies 4, 5, 6, and 7) and one OSCC group (study 4). Two OSCC groups (studies 1 and 7) were highly dominated with Bacteroidota, while one OSCC group (study 7) was dominated with Fusobacteriota (**Supplementary Figure 20A**).

At the genus-level analysis, *Streptococcus* dominated in nine healthy groups (studies 1, 2, 3, 4, 6, 8, 9, 10, and 11) and seven OSCC groups (studies 1, 2, 3, 4, 6, 10, and 11). The genus *Prevotella* showed dominance in one healthy (study 7) and two OSCC groups (studies 11 and 8). Furthermore, *Haemophilus* is abundant in the healthy group of study 5, and *Neisseria* or *Fusobacterium* is highly abundant in study 9 and study 5, respectively (**Supplementary Figure 20B**).

Species-level analysis revealed that *C. leadbetteri* were abundant in four OSCC groups (studies 1, 2, 3, and 4) as compared to the healthy group (studies 1 and 2). *C. sputigena* was found to be abundant in OSCC groups (studies 5, 8, and 9) and the healthy group (study 6). Furthermore, *P. nanceiensis* was dominant in four healthy groups (studies 3, 7, 9, and 10) and two OSCC groups (studies 7 and 10). *T. medium* showed dominance in the healthy group of study 4 and study 5 and the OSCC group of study 6 (**Supplementary Figure 20C**).

### Biomarker potential of conserved taxa in patients with OSCC

LEfSe analysis identified several discriminatory taxa between the OSCC and control groups. *Fusobacterium*, *Capnocytophaga*, *Pseudomonas*, *Porphyromonas*, *Prevotella*, *Alloprevotella*, *Filifactor*, *Gracilibacteria*, *Pseudarthrobacter*, *Serratia*, *Kingella*, and *Solibacillus* were identified as potential biomarker taxa in the OSCC group. However, 31 genera, including *Streptococcus*, *Rothia*, *Haemophilus*, *Veillonella*, *Staphylococcus*, *Lautropia*, *Acinetobacter*, *Moraxella*, *Corynebacterium*, *Delftia*, *Stenotrophomonas*, *Ralstonia*, and *Clostridia*, were recognized as potential biomarker taxa for the healthy group (**Figure 4A**). At the species level, 39 different species were identified as potential biomarkers for OSCC, showing significant differences among patients with OSCC. These include *S. infantis*, *C. leadbetteri*, *C. sputigena*, *P. bacterium*, *C. showae*, *S. sputigena*, *P. psychrophila*, *P. baroniae*, *P. saccharolytica*, and *F. frigoris*. Meanwhile, 38 species were significantly enriched in the healthy group, including *R. mucilaginosa*, *L. reuteri*, *R. dentocariosa*, *H. parahaemolyticus*, *H. sputorum*, *L. ruminis*, *P. dentalis*, and *C. durum* (**Figure 4B**). These findings were consistent with the quantitative meta-analysis, which demonstrated moderate enrichment of *Fusobacterium* and moderate depletion of *Streptococcus* across the included studies (**Supplementary Figure 20**).

**Figure 4:**
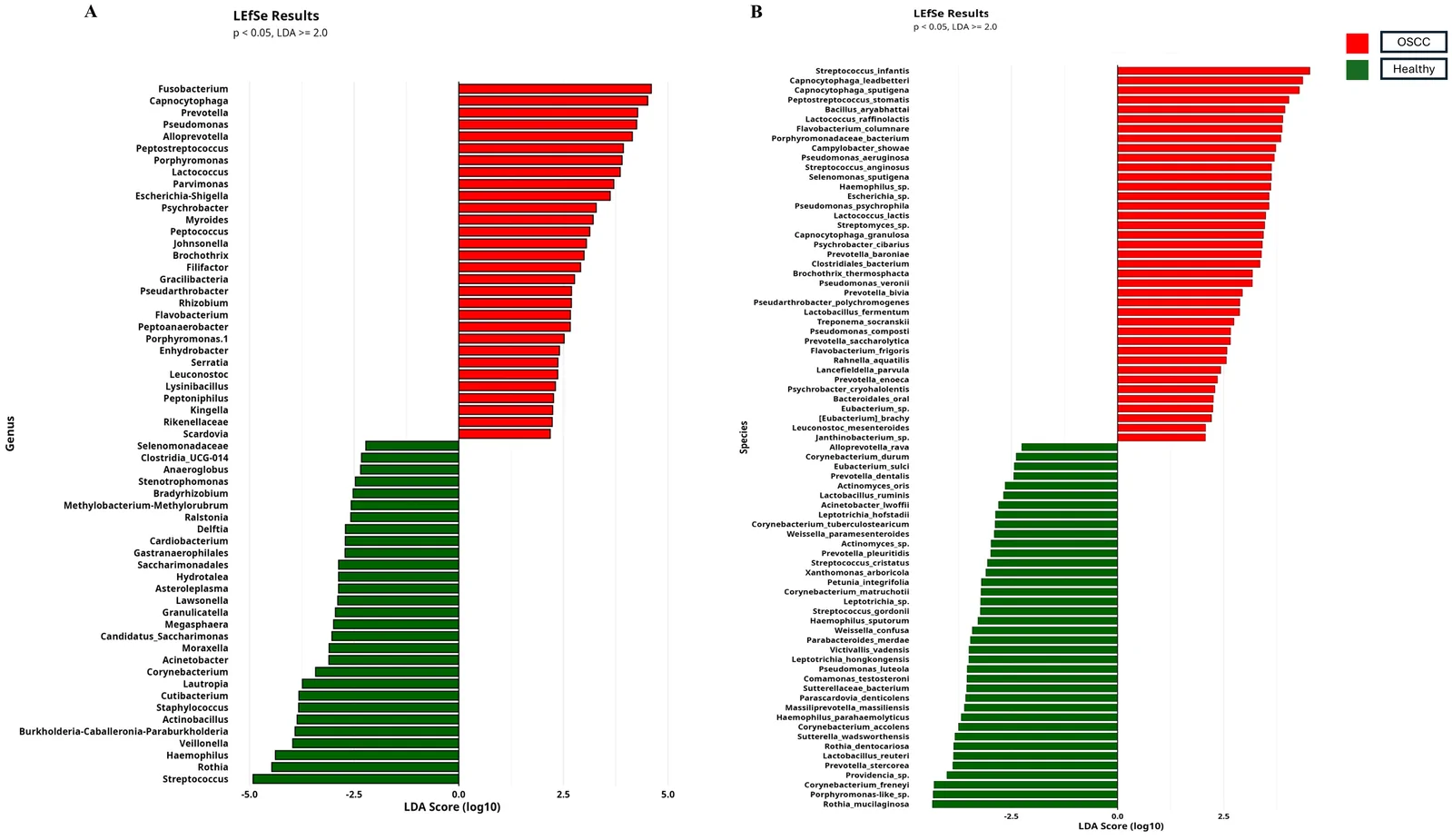
**(A)** LEfSe analysis for conserved taxa identification between OSCC (shown in red) and healthy groups (shown in green) revealed significantly differentially abundant genera. **(B)** Species-level conserved taxa identification between OSCC and healthy groups, based on LDA score ranking.

### Machine learning-based identification of key oral microbiota associated with OSCC

The LightGBM-based machine learning approach has also been used for the identification of key microbial taxa associated with OSCC, which demonstrates robust performance, achieving an AUC of 0.84 based on receiver operating characteristic (ROC) analysis (**Figure 5A**). Spearman correlation analysis of the top microbial taxa revealed distinct co-occurrence patterns among bacterial genera associated with OSCC. Strong positive correlations were observed between *Myroides* and *Brochothrix*, between *Peptostreptococcus* and *Parvimonas*, and between *Staphylococcus* and *Cutibacterium*, indicating potential cooperative interactions within the tumor-associated microbiome. However, genera including *Rothia* and *Haemophilus* showed weak to negative correlations with several anaerobic taxa, reflecting disruption of microbial homeostasis (**Figure 5B**), while Spearman correlation analysis of the top 20 species-level microbial taxa revealed distinct co-occurrence patterns within the OSCC-associated microbiome. A strong positively correlated cluster comprising *F. columnare*, *B. aryabhattai*, *Streptomyces* sp., *L. raffinolactis*, *P. cibarius*, *P. psychrophila*, *L. lactis*, and *B. thermosphacta* indicates the presence of cooperative microbial communities within the tumor microenvironment. Additionally, species such as *C. sputigena*, *C. leadbetteri*, *C. showae*, *P stomatis*, and *S. sputigena* exhibited moderate positive correlations, showing their potential involvement in OSCC-associated microbial dysbiosis. Some other species such as *R. dentocariosa*, *S. anginosus*, and *H. sputorum* demonstrated weak to negative correlations with tumor-associated taxa (**Figure 5C**).

**Figure 5:**
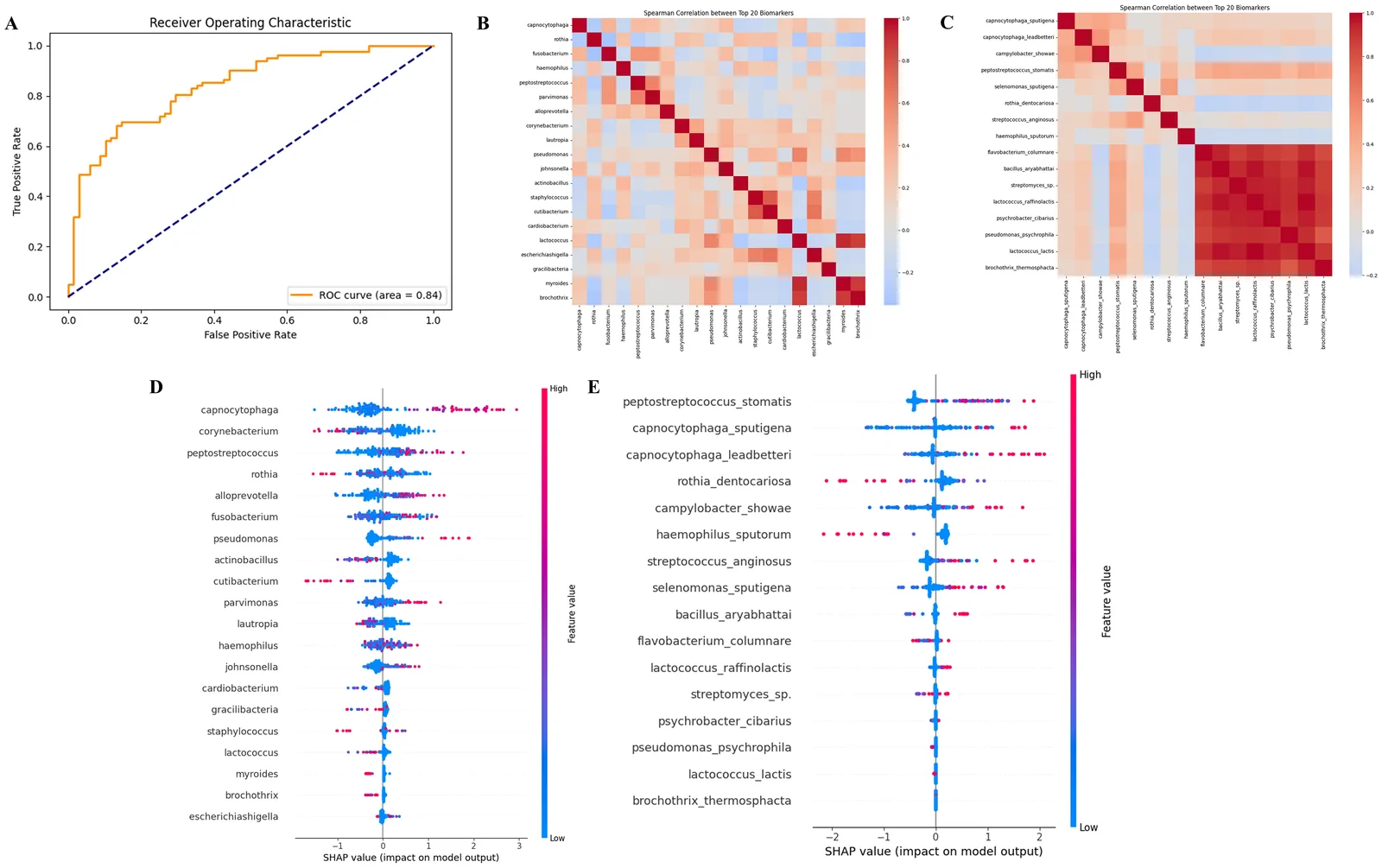
**(A)** Receiver operating characteristic (ROC) curve analysis for model validation, illustrating true positive rate (TPR) vs. false positive rate (FPR). **(B)** Heatmap represents the Spearman correlation among the top microbial taxa associated with OSCC at the genus level. **(C)** Heatmap represents the Spearman correlation among the top microbial taxa associated with OSCC at the species level. **(D)** SHAP values illustrating the contribution of each feature to individual predictions at the genus level. **(E)** SHAP values illustrating the contribution of each feature to individual predictions at the species level.

SHAP (SHapley Additive exPlanations) represents the key microbial genera contributing to classification performance between OSCC and control samples. Genera such as *Capnocytophaga*, *Corynebacterium*, *Peptostreptococcus*, and *Fusobacterium* exhibited higher SHAP values, indicating their strong contribution toward OSCC prediction, while genera including *Corynebacterium*, *Rothia*, *Haemophilus*, and *Lautropia* showed negative SHAP values, indicating their association with healthy controls and potential protective roles in oral malignancy (**Figure 5D**). SHAP analysis at the species level identified key microbial taxa contributing to OSCC classification, whereas species including *P. stomatis*, *C. sputigena*, *C. leadbetteri*, *C. showae*, and *S. sputigena* exhibited higher SHAP values, indicating their strong association with OSCC samples. Species such as *R. dentocariosa* and *H. sputorum* demonstrated negative SHAP values, indicating their association with control samples (**Figure 5E**).

### Functional prediction

Functional prediction has been carried out using PICRUSt analysis, which generates putative functional genes in four tier levels. The tier I level revealed that the majority of genes were involved in patients with OSCC, followed by healthy individuals. The Mann–Whitney comparison between OSCC and healthy individuals revealed that environmental information processing, genetic information processing, cellular processes, genetic information processing, and organismal systems showed significant differences. Additionally, the tier IV level also showed the abundance of gene involvement in the OSCC group, and their Mann–Whitney comparison at the tier IV level shows the significant difference in ABC transporters, cellular community, and cell motility between both groups (**Figure 6**).

**Figure 6:**
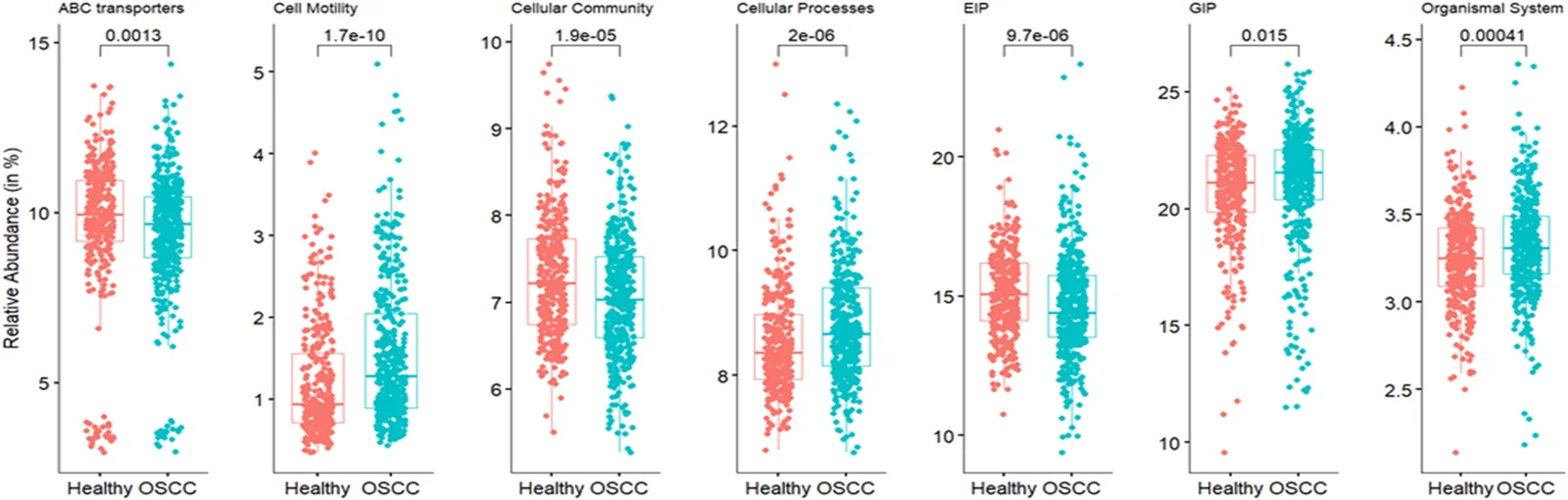
Dysregulated microbial functional pathways in OSCC. Box plots represent the differential abundance of the key functional pathways (ABC transporters, cell motility, cellular community, cellular processes, EIP* environmental information processing, GIP* genetic information processing, and organismal system) between both the healthy and OSCC groups.

Furthermore, we discussed genes involved in pro-inflammatory responses, anti-inflammatory responses, ABC transporters, and immune-related pathways for an extensive analysis at the tier IV level. A total of 150 genes were identified in pro-inflammatory pathways, of which 42 showed significant differences between the two groups. Additionally, 15 genes (*hlyE*, *hlg*, *toxA*, *stxB*, *se*, *sagA*, *sipB*, *toxR*, *cbtA*, *hlyII*, *ipgD*, *wzxC*, *ccdA*, *yopJ*, and *sopE*) exhibited Log2FC values >2 (**Figure 7A**). A total of 41 genes involved in predicted anti-inflammatory pathways were identified. Of these, 10 genes were significantly differentially abundant, with *egtE*, *egtD*, and *FLDH* exhibiting Log2FC values >2 (**Figure 7B**). In the analysis of ABC transporters, we identified a total of 532 genes, with 220 showing significant differences between the healthy and OSCC groups. Among these, 32 genes (*aguE*, *yscP*, *aguF*, *lacK*, *aguG*, *occP*, *occM*, *dgaA–D*, *yscX*, *aglK*, *glnH*, *thuF*, *aotJ*, *occT*, *natA–B*, *occQ*, *yydJ*, *yydI*, *stp1*, *iroC*, *aotP*, *aotQ*, *aotM*, *thuE*, *somE*, *yscW*, and *smoF–G*) had Log2FC values >2 (**Figure 7C**) and only 85 genes were identified in immune-related pathways, with 28 showing significant differences. Out of these, only one gene (*ccdA*) displayed Log2FC values >2 (**Figure 7D**).

**Figure 7:**
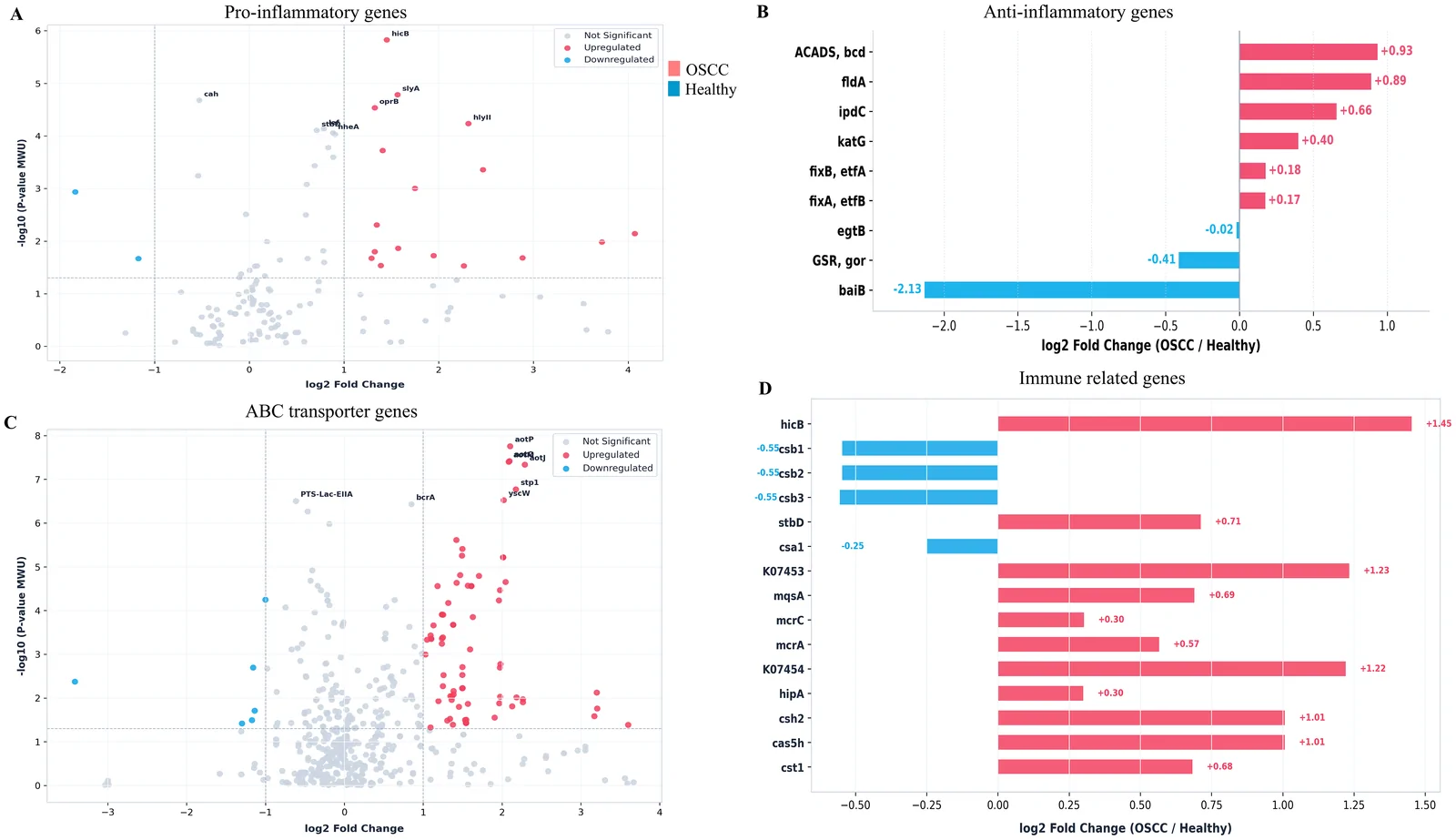
Volcano plots showing the differential abundance of pro-inflammatory genes **(A)** and ABC transporter genes **(C)** between healthy controls and patients with OSCC, based on log2 fold change and Mann–Whitney *U* test-derived *p*-values. Bar plot illustrating the differential abundance of anti-inflammatory genes **(B)** and bacterial immune-related genes **(D)** between the study groups, highlighting significantly enriched and depleted functional markers.

### Network analysis connecting dysbiotic taxa to inflammatory signatures, bacterial transporters, and bacterial immunity

Spearman’s rank correlation analysis revealed extensive associations between differentially abundant bacterial genera and predicted pro-inflammatory genes (**Figure 8**). The correlation network was primarily characterized by positive associations between virulence-associated genes and oral bacterial taxa. Among these, *Paraburkholderia* exhibited maximal positive correlations with the hemolysin toxin regulator *fitG* (ρ = 0.469) and the virulence regulator *tcpI* (ρ = 0.467). Similarly, *Porphyromonas* also showed a positive correlation with several virulence-associated genes, including the adenylate cyclase toxin *cyaA* (ρ = 0.391), the lethal factor *lef* (ρ = 0.388), and the non-hemolytic enterotoxin genes *nheA* (ρ = 0.385) and *nheBC* (ρ = 0.384). In contrast, *Clostridia_UCG-014* demonstrated a negative correlation with the pro-inflammatory toxin transporter component *ABC-2.CYL* (ρ = −0.388). Additionally, predicted anti-inflammatory genes revealed multiple significant associations between bacterial genera and genes involved in bile acid metabolism, tryptophan metabolism, oxidative stress response, and short-chain fatty acid metabolism. *Methylobacterium–Methylorubrum* showed maximal positive correlation with the bile acid-CoA ligase gene *baiB* (ρ = 0.400), followed by *Hydrotalea* (ρ = 0.313) and *Bradyrhizobium* (ρ = 0.277). Moreover, *Lactococcus* was positively correlated with the ROS-scavenging catalase gene *katE* (ρ = 0.277). Conversely, *Porphyromonas* exhibited a negative correlation with *ipdC*, encoding indolepyruvate decarboxylase involved in indole biosynthesis (ρ = −0.295). Similarly, *Clostridia_UCG-014* was negatively correlated with the propionate metabolism gene *MCEE/epi* (ρ = −0.282) and the electron transfer flavoprotein genes *fixB/etfA* (ρ = −0.274) and *fixA/etfB* (ρ = −0.274) (**Supplementary Figure 21**).

**Figure 8:**
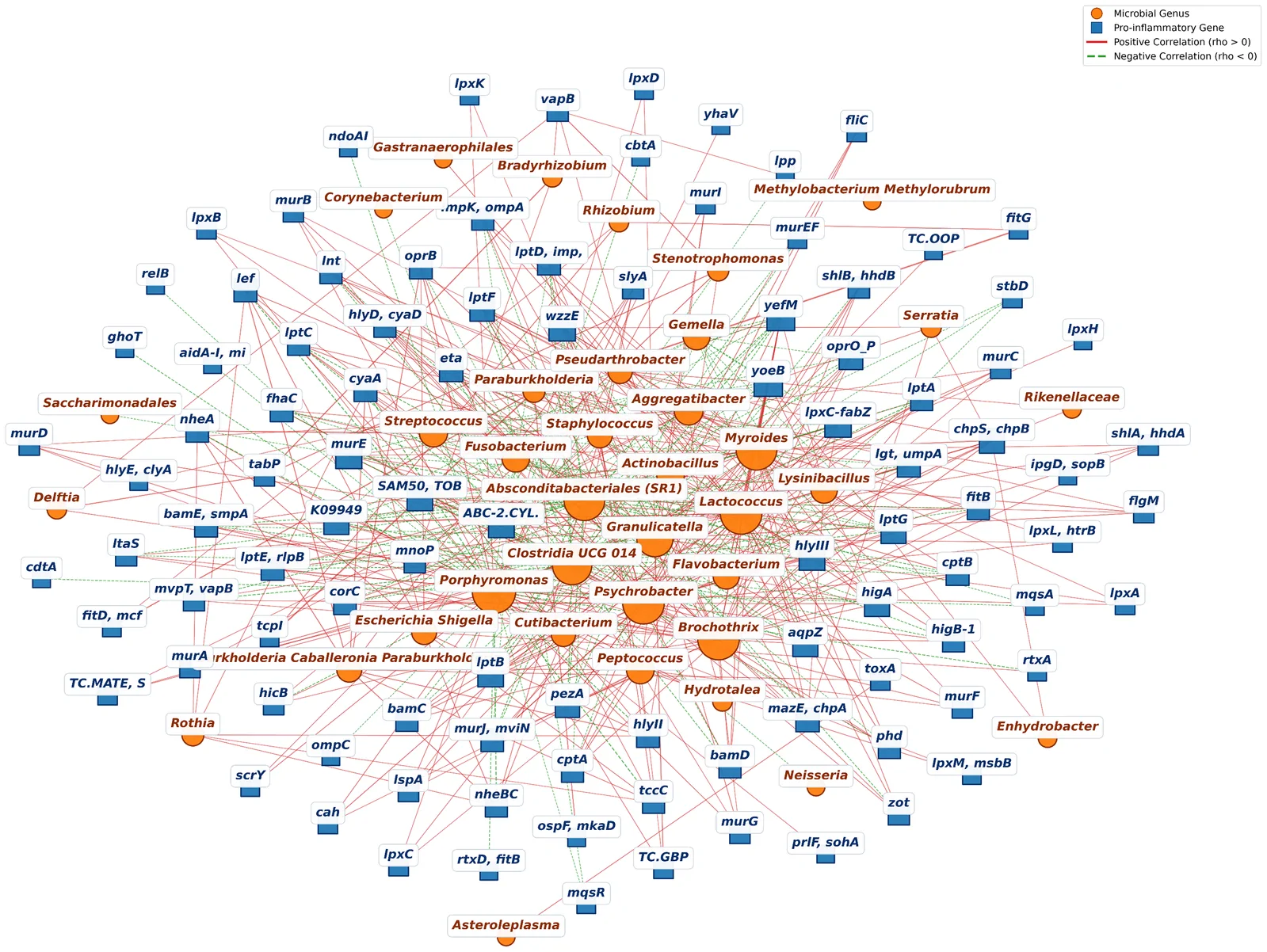
Correlation network analysis of bacterial genera significantly associated with predicted microbial pro-inflammatory functional genes.

Spearman’s rank correlation analysis of predicted ABC transporter genes revealed significant positive associations between differentially abundant bacterial genera and genes involved in nutrient transport. *Escherichia-Shigella* was positively correlated with the riboflavin transporter component *yscW* (ρ = 0.273) and the general iron/sugar transporter *stp1* (ρ = 0.267). Similarly, *Cutibacterium* exhibited positive correlations with *stp1* (ρ = 0.273), *yscW* (ρ = 0.267), and the iron-complex transport system proteins *aotQ* (ρ = 0.247) and *aotP* (ρ = 0.242). In addition, *Staphylococcus* showed a positive correlation with the nutrient transporter *yscW* (ρ = 0.268). Overall, the correlation network demonstrated extensive positive associations between bacterial genera and predicted ABC transporter genes, suggesting coordinated variation in microbial taxa and nutrient transport-related functional potential (**Supplementary Figure 22**). However, predicted bacterial immunity-related genes revealed distinct patterns of association between differentially abundant bacterial genera and immune-associated genes. *Lactococcus* exhibited maximal positive correlations with the cold-shock protein genes *csb1* (ρ = 0.365), *csb2* (ρ = 0.365), and *csb3* (ρ = 0.351). Similarly, *Psychrobacter* and *Brochothrix* showed positive correlations with *csb2* (ρ = 0.337) and *csa1* (ρ = 0.329 for *Psychrobacter*; ρ = 0.324 for *Brochothrix*). Furthermore, *Clostridia_UCG-014* exhibited a negative correlation with the toxin–antitoxin system component *yefM* (ρ = −0.324). Overall, the correlation network demonstrated both positive and negative associations between bacterial genera and predicted immunity-related genes (**Figure 9**).

**Figure 9:**
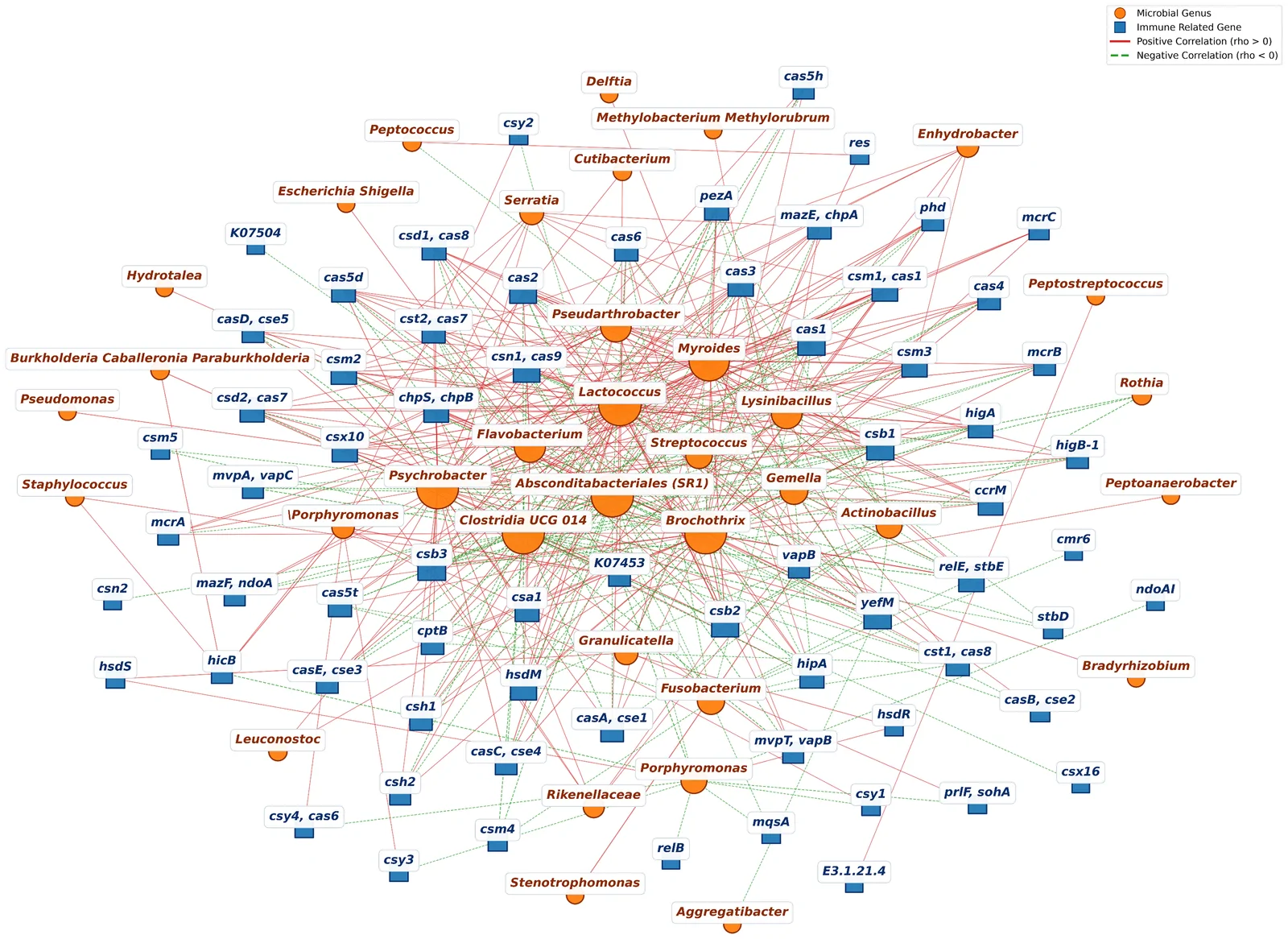
Correlation network analysis of bacterial genera significantly associated with predicted microbial immune-related functional genes.

## Discussion

The current study reveals that oral microbes may associate with the progression of OSCC, and specific characteristics of bacterial alterations can be used as biological signatures for the disease. Some bacterial phyla, such as Bacteroidota, Firmicutes, Fusobacteriota, Proteobacteria, and Spirochaetota, were significantly high in OSCC groups while two different phyla, Actinobacteriota and Cyanobacteria, were significantly high in healthy groups. Similarly, the phylum Firmicutes, followed by Actinobacteria, Fusobacteria, Bacteroidetes, and Proteobacteria, were reported in both the healthy group and patients with OSCC, but Fusobacteria, Bacteroidetes, and Proteobacteria were comparatively high in OSCC groups (17, 23). Firmicutes and Bacteroidetes were also enriched in the salivary microbiome of both patients with OSCC and those with head and neck cancer (24). High abundance of Bacteroidetes, Fusobacteria, and Proteobacteria in OSCC samples can be correlated with inflammation-associated change in microbiome composition (25). *Porphyromonas*, *Fusobacterium*, *Campylobacter*, *Catonella*, *Filifactor*, *Peptoaerobacter*, *Prevotella*, and *Treponema* were significantly enriched in patients with OSCC at the genus level. *Neisseria*, *Gemella*, *Rothia*, *Actinobacillus*, *Corynebacterium*, *Gracilibacteria*, *Haemophilus*, and *Lautropia* were significantly abundant in healthy groups. However, genera *Mycoplasma*, *Lautropia*, *Campylobacter* and *Leptotrichia* were significantly abundant in OSCC samples as compared to the healthy group (26). *Fusobacterium*, *Alloprevotella*, *Campylobacter*, and *Porphyromonas* were enriched in cancer tissues, which can induce chronic inflammation, produce carcinogenic metabolites, and contribute to malignancy (27, 28). *Prevotella*, for instance, potentially influences oncogenic processes through mechanisms like biofilm formation (10). *Pseudomonas*, *Capnocytophaga*, *Mycoplasma*, and *Peptostreptococcus* were associated with OSCC, while *Actinobacillus*, *Veillonella*, and *Corynebacterium* act as potential biomarkers for the healthy population (17). The enrichment of *Capnocytophaga*, *Fusobacterium*, *Leptotrichia*, *Neisseria*, and *Bergeyella* in patients with OSCC has been widely reported (29, 30). The genus *Filifactor* was less reported in other studies except for one that observed a significant positive correlation with other genera in tumor sites (31). *Filifactor* also acts as a potential biomarker in the tobacco chewer group (17). The genus *Haemophilus* was highly abundant in healthy groups from Yemen, whereas two other studies reported that *Haemophilus* is highly dominant in OSCC groups of the Indian cohort (15, 23). *Streptococcus*, *Neisseria*, *Prevotella*, *Porphyromonas*, *Haemophilus*, and *Veillonella* are also highly dominant in healthy individuals (15, 17, 25).

Species-level taxonomic resolution reveals the dominance of *C. showae*, *P. intermedia*, *P. melaninogenica*, *Selenomonas* sp., *S. sputigena*, *S. pneumoniae*, *S. anginosus*, and *C. leadbetteri* in OSCC groups. *In vitro* heat-killed *P. intermedia* alters the tumor microenvironment and promotes OSCC progression, by suppressing tumor suppressor genes and upregulating the expression of pro-inflammatory cytokines in OSCC tissues (32). *C. showae* is present as a prophylactic biomarker in oral microbiome research, as it increases the susceptibility to OSCC and HNSCC and correlates with a 50% higher cancer risk (33). Notably, *P. gingivalis* and *F. nucleatum* are involved in most pathways related to OSCC development. Additionally, various inflammatory cytokines, such as IL-6, IL-8, TNF-α, and PGE, affect the tumor microenvironment (34). In this meta-analysis, four studies showed the abundance of *P. gingivalis* in ≥0.5% (26, 32, 35, 36). *S. anginosus* acts as a potential biomarker as it frequently increases in patients with OSCC (37). Other species, such as *H. parainfluenzae*, *R. aeria*, *R. dentocariosa*, and *R. mucilaginosa*, were inhibited in the OSCC group in the study, while *R. mucilaginosa* was previously reported as a community member in cystic fibrosis, where bacterial iron cycling plays a major role in virulence development (38). *R*. *mucilaginosa* is the most dominant species among all *Rothia* species in the oral cavity of humans in the Japan cohort (39). *Rothia dentocariosa* can have virulence factors that can contribute to pathogenic potential and be linked to OC and oral dysbiosis (40). *H. parainfluenzae*, present as an independent species and more common in the human oral cavity (41), is highly dominant in healthy controls (23). *H. parainfluenzae* also reduces the level of Sjogren’s syndrome and elevates homeostatic T-cell immunity (42). Meanwhile, some other reports favor *Haemophilus* species acting as a prognostic biomarker, as they are enriched in patients with OSCC with favorable survival outcomes, and they are also associated with immune-related transcriptional activities (43) (15),. Furthermore, *S. moorei* were highly abundant in the OSCC group. This microorganism promotes tumor progression through the integrin α2/β1-PI3K-AKT-mTOR-C-myc signaling pathway, which acts as a new potential target for the prevention, diagnosis, and treatment of colorectal cancer (CRC) (Chen et al., 2025).

*C. sputigena*, *Haemophilus* sp., and *M. salivarium* were found to be enriched in the OSCC group in India. This finding aligns closely with an earlier study that also noted significant increases in *Haemophilus*, *Capnocytophaga*, *Porphyromonas*, *Treponema*, and *C. gingivalis* in OSCC cases (44). Species such as *C. showae*, *C. sputigena*, *P. stomatis*, *P. abscessus*, *P. baroniae*, *P. intermedia*, *P. loescheii*, *P. melaninogenica*, *S. anginosus*, *T. denticola*, and *T. lecithinolyticum* were significantly enriched in Chinese patients. Furthermore, there were notable enrichments of *Fusobacterium, Dialister, Filifactor, Prevotella, Catonella*, and *Parvimonas* in OSCC samples, as reported by Zhao et al. (45). The genus *Lautropia* was commonly observed across various countries, including India, Yemen, China, and Taiwan. Additionally, a separate study from Taiwan reported that species such as *S. constellatus*, *H. influenzae*, and *F. alocis* increased progressively in patients with OSCC, while levels of *Streptococcus*, *Haemophilus*, and *Porphyromonas* decreased as cancer progressed (46). In terms of beta-diversity, it showed a significant difference between healthy and OSCC groups (Bray–Curtis SMD = 1.29, 95% CI: 0.68–1.90; *I*² = 81.8%), while alpha-diversity results were inconsistent across studies. Some studies reported no significant alpha-diversity differences (47), whereas others found higher Simpson and Shannon indices in patients with cancer (36).

The computational analysis of functional pathways showed that cell motility, environmental adaptation, metabolism, and all tier I pathways were enriched in the OSCC group. Similarly, pathways related to cancer, cell motility, environmental adaptation, metabolism, and signal transduction were highly abundant in the tumor group (30). Further predictions at the tier IV level indicated an upregulation of ABC transporter genes in the OSCC group, which can serve two important functions: they import essential nutrients and export toxic molecules (48). ABC transporters have two notable roles: they import essential nutrients and export toxic molecules (48). Genes involved in bacterial chemotaxis, flagellar assembly, and lipopolysaccharide (LPS) biosynthesis were significantly present in the OSCC group, which could be considered diagnostic markers (25, 49, 50). The predictive pathways (cell adhesion, EMT, and proliferation) were also reported in a transcriptomic analysis of OSCC samples, which were associated with enriched pathobionts (*T. medium*, *P. stomatis*, and *C. morbi*) through epigenetic modulation (19). *P. intermedia* is a well-known pathogen associated with periodontitis, capable of secreting peptides and proteases (51, 52). The proteases function as signaling molecules by activating proteinase-activated receptors (PARs), which are involved in critical processes such as cell proliferation, apoptosis, autoimmunity, cytokine production, microenvironmental inflammation, pain signaling, and epithelial barrier regulation (53, 54). Bacterially derived proteases can degrade host components such as the extracellular matrix (ECM), disrupt physical barriers, and modulate immune responses, which may ultimately contribute to tumor initiation and progression (55). The bacterial inflammatory processes, including LPS synthesis, flagellar assembly, and bacterial chemotaxis, have been implicated in mediating the inflammation associated with cancer development (23, 34). LPS is recognized by LPS-binding protein and Toll-like receptor 4 (TLR4), which activates the cytokine transcription through the detection of pathogen-associated molecular patterns (PAMPs) (56). The majority of microbial activity drives chronic inflammation in the oral cavity, which is observed at various stages of OSCC (46, 57). The significant enrichment of pro-inflammation-related genes such as *hlyE*, *hlg*, *toxA*, *stxB*, *se*, *sagA*, *sipB*, *toxR*, *cbtA*, *hlyII*, *ipgD*, *wzxC*, *ccdA*, *yopJ*, and *sopE* in the OSCC group is associated with toxins, secretion system effectors, and hemolysins. These genes are known to promote host cell damage, modulate the immune response, and support bacterial persistence. Similar patterns of virulence-associated functions have been observed in OC microbiomes, where an increased abundance of genes related to bacterial invasion, motility, and pathogenicity correlates with tumor progression and poorer clinical outcomes (46, 58). In addition to their roles in virulence and pro-inflammatory responses, the functional predictions identified changes in genes associated with anti-inflammatory pathways. Among these, *egtE* and *egtD*, which are involved in the biosynthesis of ergothioneine, as well as *FLDH*, associated with formaldehyde detoxification and cellular redox homeostasis, showed maximum enrichment. Ergothioneine is a sulfur-containing antioxidant produced by bacteria and fungi that helps protect cells from ROS, oxidative stress, and redox imbalances (59, 60). Likewise, formaldehyde dehydrogenase plays a role in detoxifying reactive aldehydes produced during cellular metabolism and oxidative stress, thus aiding microbial survival in hostile environmental conditions (61). Moreover, the presence of transporter genes such as *aguE*, *aguF*, *aguG*, *yscP*, *aotQ*, *aotM*, *thuE*, *occP*, *occM*, *occQ*, and *iroC* plays a crucial role in nutrient acquisition, metabolite transport, stress adaptation, and bacterial survival in complex host environments. Studies have shown that various transporter systems are characteristic of cancer-associated microbiomes, enabling adaptation to the nutrient-rich and inflammatory environment of tumors (35, 62). The immune-related gene *ccdA* is part of a toxin–antitoxin system that enhances bacterial stress tolerance and persistence under adverse environmental conditions. Bacterial immunity, such as toxin–antitoxin systems, stress-response pathways, and biofilm formation, enhances microbial survival under immune-mediated stress and supports long-term colonization. Persistent microorganisms continuously activate inflammatory signaling pathways, including NF-κB and STAT3, which lead to an increased production of pro-inflammatory cytokines, oxidative stress, and tissue damage. Additionally, microbial adaptation and immune evasion strategies can inhibit antitumor immune responses and foster a tumor-supportive microenvironment, thereby contributing to the initiation and progression of cancer (63). Intratumoral microbial abundance influences the immune microenvironment of OSCC by affecting immune cell infiltration and immune-related signaling, further supporting the role of host–microbiome interactions in tumor progression (64). The significant enrichment of bacterial genes involved in inflammation, immune-related functions, and virulence or transporters are underlying mechanisms for bacterial persistence and alteration of host pathways within the tumor microenvironment. Similar findings have shown that OC-associated microbiota tend to exhibit functions related to persistence and immune evasion, rather than directly activating host immune pathways (62, 65).

This study presents a comprehensive meta-analysis integrating datasets from diverse geographic regions and sample types through combined taxonomic and functional analyses. This integrative approach enhances the robustness of the identified microbial signatures and predicted functional pathways associated with OSCC, providing valuable insights into potential diagnostic and prognostic biomarkers. However, the study is subject to substantial heterogeneity arising from differences in sample types, population characteristics, lifestyle factors, sequencing methodologies, and the uneven geographic representation of the included studies. The predicted functional reprogramming suggests an increased potential for bacterial colonization, persistence, chronic inflammation, and tumor progression, further supporting the concept that microbial functional dysbiosis, rather than taxonomic composition alone, may play an important role in oral carcinogenesis. These findings provide a foundation for future investigations into microbiome-based biomarkers and targeted therapeutic strategies. Nevertheless, the observed taxonomic and functional alterations represent associations rather than causal relationships. The cross-sectional design of the included datasets precludes determination of whether microbial dysbiosis precedes tumor development or arises as a consequence of tumor-associated changes in the oral microenvironment. Although previous experimental studies have proposed plausible mechanisms linking oral microorganisms with chronic inflammation, immune modulation, and carcinogenesis, well-designed longitudinal studies, microbiome intervention trials, and causal inference approaches are required to establish the temporal and causal role of the oral microbiome in OSCC.

## Conclusion

This study demonstrates that microbes may play an associative role in the progression of OSCC. Several bacterial species, including *C. sputigena*, *L. bacterium*, *Haemophilus* sp., *P. loescheii*, *S. anginosus*, *S. pneumoniae*, and *T. denticola*, have been identified as potential biological indicators for OC. The transition from a healthy oral microbiome to a dysbiotic bacteriome can be associated with chronic inflammation and may activate cancer-promoting pathways. The variations in microbial composition across different geographical regions, such as the presence of *C. sputigena*, *Haemophilus* sp., and *M. salivarium* in Indian cohorts versus *C. showae*, *T. denticola*, and *P. intermedia* in Chinese populations, suggest that lifestyle, genetics, and environmental factors can influence regional microbiome differences. Beyond taxonomic dysbiosis, the study demonstrates that OSCC is associated with alterations in predicted pro-inflammatory, anti-inflammatory/stress-response, and ABC transporter pathways, indicating enhanced bacterial adaptation, persistence, and potential within the tumor microenvironment. Along with immune-related pathways, microbial survival and virulence mechanisms may play a role in oral carcinogenesis. These findings highlight that changes in oral microbial communities could serve as potential diagnostic and prognostic biomarkers. They may appear as therapeutic targets or adjuvants with standard therapeutic regimens for OSCC. Further longitudinal and multi-omics studies are warranted to validate these findings and elucidate causal relationships between microbial dysbiosis and oral carcinogenesis.

## Data Availability

The original contributions presented in the study are included in the article/**Supplementary Material**. Further inquiries can be directed to the corresponding authors.
